# An *In-vivo* 1H-MRS short-echo time technique at 7T: Quantification of metabolites in chronic multiple sclerosis and neuromyelitis optica brain lesions and normal appearing brain tissue

**DOI:** 10.1016/j.neuroimage.2021.118225

**Published:** 2021-09

**Authors:** George Tackley, Yazhuo Kong, Rachel Minne, Silvia Messina, Anderson Winkler, Ana Cavey, Rosie Everett, Gabriele C DeLuca, Andrew Weir, Matthew Craner, Irene Tracey, Jacqueline Palace, Charlotte J Stagg, Uzay Emir

**Affiliations:** a Wellcome Centre for Integrative Neuroimaging, FMRIB, Nuffield Department of Clinical Neurosciences, University of Oxford, Oxford, OX3 9DU, United Kingdom; b Cardiff University Brain Research Imaging Centre (CUBRIC), Cardiff University, CF24 4HQ, United Kingdom; c CAS Key Laboratory of Behavioural Science, Institute of Psychology, Chinese Academy of Sciences, Beijing 100101, China; d Department of Psychology, University of Chinese Academy of Sciences, Beijing 100049, China; e School of Health Sciences, Purdue University, 550 Stadium Mall Drive, West Lafayette, IN 47907, (765) 494-1419, United States; f Weldon School of Biomedical Engineering, Purdue University, West Lafayette, IN, United States; g Division of Clinical Neurology, Nuffield Department of Clinical Neurosciences, University of Oxford, Oxford, OX3 9DU, United Kingdom; h National Institute of Mental Health (NIMH), National Institutes of Health (NIH), Bethesda, MD, United States; i MRC Brain Network Dynamics Unit, University of Oxford, Oxford, OX1 3TH, United Kingdom

**Keywords:** Neuroinflammatory disorders, Multiple Sclerosis, Neuromyelitis optica, Magnetic resonance spectroscopy, Ultra-high field MRI, Ala, L-Alanine, Asc, ascorbate, Asp, Aspartate, Cr, creatine, GABA, gamma aminobutyric acid, Glc+Tau, glucose and taurine, Gln, glutamine, Glu, glutamate, Glx, Gln + Glu, GPC, glycerophosphocholine, GSH, glutathione, Ins, *myo*-inositol, Lac, L-lactate, mm, macromolecules, NAA, N-acetylaspartate (i.e. *not* including NAAG), NAAG, N-acetylaspartylglutamate, PCho, Phosphocholine, PCr, phosphocreatine, PE, phosphorylethanolamine, Scyllo, *scyllo*-Inositol, Tau, taurine, tCho, total choline (GPC + Cho), tCr, total creatine (Cr + PCr), tNAA, total N-acetylaspartate (NAA + NAAG)

## Abstract

Magnetic Resonance Spectroscopy (MRS) allows for the non-invasive quantification of neurochemicals and has the potential to differentiate between the pathologically distinct diseases, multiple sclerosis (MS) and AQP4Ab-positive neuromyelitis optica spectrum disorder (AQP4Ab-NMOSD). In this study we characterised the metabolite profiles of brain lesions in 11 MS and 4 AQP4Ab-NMOSD patients using an optimised MRS methodology at ultra-high field strength (7T) incorporating correction for T2 water relaxation differences between lesioned and normal tissue.

MS metabolite results were in keeping with the existing literature: total N-acetylaspartate (NAA) was lower in lesions compared to normal appearing brain white matter (NAWM) with reciprocal findings for *myo*-Inositol. An unexpected subtlety revealed by our technique was that total NAA differences were likely driven by NAA-glutamate (NAAG), a ubiquitous CNS molecule with functions quite distinct from NAA though commonly quantified together with NAA in MRS studies as total NAA. Surprisingly, AQP4Ab-NMOSD showed no significant differences for total NAA, NAA, NAAG or *myo*-Inositol between lesion and NAWM sites, nor were there any differences between MS and AQP4Ab-NMOSD for *a priori* hypotheses. Post-hoc testing revealed a significant correlation between NAWM Ins:NAA and disability (as measured by EDSS) for disease groups combined, driven by the AP4Ab-NMOSD group.

Utilising an optimised MRS methodology, our study highlights some under-explored subtleties in MRS profiles, such as the absence of *myo*-Inositol concentration differences in AQP4Ab-NMOSD brain lesions versus NAWM and the potential influence of NAAG differences between lesions and normal appearing white matter in MS.

## Highlights

• NAAG likely contributes to the total NAA differences between multiple sclerosis lesion and normal appearing brain tissue.
• *myo*-Inositol was *not* shown to be different between chronic AQP4Ab-NMOSD brain lesions and normal appearing brain tissue.
• An optimised MRS methodology is described, using 7T field strength and correcting for tissue T2 water relaxion differences.
• 7-tesla MRS profiles of chronic brain lesions and normal appearing white matter are presented for MS and AQP4Ab-NMOSD.

## Graphical abstract

**Figure:**
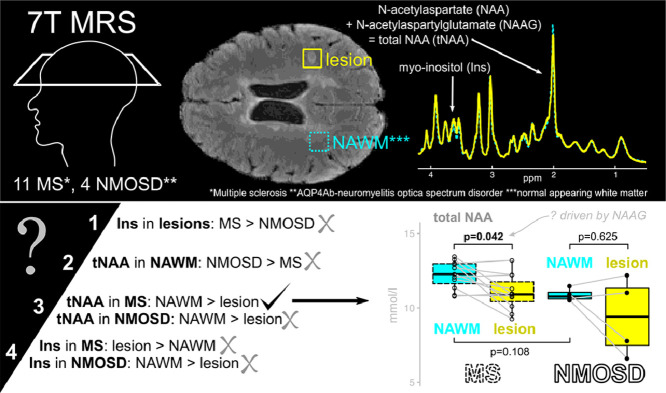

## 1 Introduction

Magnetic Resonance Spectroscopy (MRS) allows for the non-invasive quantification of neurochemicals, and therefore has the potential to differentiate between pathologically distinct diseases. This is perhaps especially important in circumstances where clinical syndromes may overlap but treatment strategies are distinct. In particular, there is interest in utilising MRS to differentiate the primary astrocytopathy Aquaporin-4 Antibody positive Neuromyelitis Optica Spectrum Disorders (AQP4Ab-NMOSD) (Fujihara, 2011) from the clinically similar but pathologically distinct disorder, Multiple Sclerosis (MS), which is believed to be a chronic inflammatory demyelinating disorder with secondary neurodegeneration (Lucchinetti et al., 2014; Wingerchuk et al., 2015). In addition, where MRS metrics prove sensitive to the underlying pathology in a disease state, they can inform our understanding of that pathology, and potentially be developed as biomarkers for future pharmacological studies.

MRS studies in MS consistently report a core pattern of findings. Compared to healthy control brain tissue, lesions of all ages show reduced total N-acetyl aspartate (tNAA) likely reflecting decreased neuronal mitochondrial activity, and raised *myo*-Inositol-containing compounds (Ins), commonly taken to reflect glial activity (Brex et al., 2000; Ciccarelli et al., 2013; Miller et al., 2003). A similar pattern of changes is found in MS normal appearing white matter (NAWM) versus healthy controls, though to a smaller degree (De Stefano et al., 2007; Fernando et al., 2004; Helms et al., 2000; Miller et al., 2003). In addition to changes in specific metabolites, the relative concentrations of some MRS metabolites have also been correlated with clinical metrics. The relative concentration of *myo*-Inositol and tNAA in NAWM (Ins:tNAA), which provides an insight into the relative activity of glia and neurons within a given region, has been shown to predict disease progression in MS. It has been hypothesised to be a sensitive index that encapsulates both the damaging immune-mediated gliosis (increase in *myo*-Inositol) and the disabling neurodegenerative axonal loss (reduction in NAA) that are known features of MS pathology (Llufriu et al., 2014; Miller DH, 2014).

In patients with AQP4Ab-NMOSD, there is little or no evidence for a difference in either tNAA (i.e. NAA + NAAG) or *myo*-Inositol in normal appearing white matter compared to healthy controls (Aboul-Enein et al., 2010; Bichuetti et al., 2008; Kremer et al., 2015), raising the possibility that *myo*-Inositol and tNAA may be sensitive discriminators between AQP4Ab-NMOSD and MS. However, no studies to date have studied MRS-quantified neurochemicals within AQP4Ab-NMOSD lesions in the brain. A single study has examined MRS-derived neurochemical profiles in AQP4Ab-NMOSD lesions within the spinal cord and demonstrated significantly lower *myo*-Inositol in AQP4Ab-NMOSD lesions compared with both MS cord lesions and healthy controls (Ciccarelli et al., 2013), consistent with previously observed astrocyte damage and loss in AQP4Ab-NMOSD lesions. Ciccarelli *et al.* also demonstrated, in line with existing literature, a substantial decrease in tNAA in MS lesions compared with healthy controls, but no significant difference in tNAA between AQP4Ab-NMOSD lesions and either MS lesions or healthy controls.

Here, we wished to see whether MRS could distinguish between AQP4Ab-NMOSD and MS lesions in the brain. However, there are a number of technical difficulties in performing MRS in the context of neurological disease that are important to overcome in order to accurately address this question. In particular, lesioned tissue has a longer T2 than non-lesioned tissue, due to its greater water content (Zimmerman et al., 1986), which, if not compensated for (e.g. by using a short TE and T2* measurement), will directly influence metabolite quantification. In addition, it is important to fully separate neurochemicals with highly similar spectral resonances but disparate physiological functions, for example NAA and the closely chemically related, but functionally distinct, NAA-glutamate (NAAG), often combined in the MRS metric tNAA. This is particularly important in the context of neurodegeneration, as NAA reflects neuronal mitochondrial function, whereas NAAG acts as a neuromodulator (Baslow, 2000; Birken and Oldendorf, 1989; Neale et al., 2000). It is therefore difficult to clearly interpret changes in the tNAA metric in terms of the underlying pathology.

We therefore exploited recent advances in MRS methodology to study pathological differences between AQP4Ab-NMOSD and MS lesions. We used an ultra-high field (7T) scanner, to allow separation between NAA and NAAG, something not easily achievable at 3T, and modelled multiple water T2-relaxation times to compensate for lesion-related effects on metabolite quantification (Helms, 2001), approaches that have been little used in this context before now.

We wished to test the hypotheses that (1) *myo*-Inositol would be higher in MS brain lesions than AQP4Ab-NMOSD brain lesions, reflecting the likely increased astrocytic damage in AQP4Ab-NMOSD compared to the reciprocal astrogliosis found in MS lesions, and (2) that NAA (tNAA, NAA & NAAG) would be higher in the normal appearing white matter in AQP4Ab-NMOSD patients compared with MS patients, in line with the relative lack of extra-lesional neurodegeneration in AQP4Ab-NMOSD (Matthews et al., 2015). Within diseases we also hypothesised that (3) NAA (tNAA, NAA & NAAG) would be greater in NAWM than lesion sites in line with expected neuronal loss in lesions, and that (4) *myo*-inositol would be differentially *greater* in MS brain lesion versus NAWM sites and *lower* in AQP4Ab-NMOSD lesion versus NAWM sites reflecting the contrasting gliotic and astrocytopathic nature of lesions in these two conditions (see Box 1 for summary of hypotheses).

## 2 Methods

### 2.1 Subjects

Eleven patients with clinically diagnosed relapsing-remitting multiple sclerosis (RRMS) and four patients with AQP4Ab-NMOSD gave their written informed consent to participate in the study, under local ethics board approval (Oxfordshire REC A 10/H0604/99; Berkshire REC 13/SC/0238). A twelfth multiple sclerosis patient was scanned but later excluded as found to fit secondary-progressive multiple sclerosis diagnostic criteria. In addition to MR scanning, patients underwent a short clinical consultation and neurological examination including EDSS scoring. Current medications were recorded. Minimum lesion age was calculated as the time from the oldest clinical brain MRI to contain the targeted lesion, and only lesions >3 months old were included.

### 2.2 MR acquisition

MR was performed on a 7T Siemens MAGNETOM system (Siemens, Erlangen, Germany) equipped with a Nova Medical 32 channel receive array head coil. Two MRS volumes-of-interest (VOIs) were acquired per subject: one targeting a chronic, T2-hyperintense, T1-hypointense white matter lesion (>3 months old, confirmed on historical clinical MRIs) and another centred on a contralateral area of normal appearing white matter (NAWM; a majority white-matter voxel, avoiding as much grey matter and cerebrospinal fluid as possible), positioned as close as possible to contralateral reflection-symmetrical with the lesion voxel. The spectroscopy voxel volume was 15 × 15 × 15 mm^3^. Dielectric pads were not used.

Spectroscopy voxels were manually positioned by reference to a 3-dimensional 1 mm isotropic T2-weighted fluid-attenuated inversion recovery (FLAIR) image (1 mm isotropic, TR = 5 s, TE = 272 ms, TI = 1.8 s) (See Supplemental Figure 1 for individual voxel locations). First- and second-order shims were first adjusted by gradient-echo shimming (Shah et al., 2009). The second step involved only fine adjustment of first order shims using FASTMAP (Gruetter and Tkáč, 2000). Spectra were acquired using a Stimulated Echo Acquisition Mode (STEAM) pulse sequence (TE=11 ms, TR=5 s, number of transients=64) with variable power radiofrequency pulses with optimized relaxation delay (VAPOUR), water suppression and outer volume saturation (Emir et al., 2012). Unsuppressed water spectra acquired from the same voxel were used to remove residual eddy current effects and to reconstruct the phased array spectra (one transient per echo). Metabolite T2-relaxations were not calculated as these would have pushed scan time beyond the feasible limit for our clinical group with comorbid disability and pain.

Finally, fully relaxed unsuppressed water signals were acquired at TEs ranging from 11 to 4000 ms (TR=15 s) to estimate the cerebrospinal fluid (CSF) contribution to each VOI (see below). A whole-brain 3-dimensional T1-MPRAGE (1 mm isotropic, TR = 2.2 s, TE = 282 ms) was also acquired to evaluate T1 hypo-intensity in lesions and used in conjunction with T2-weighted images to (1) aid manual segmentation of lesions and (2) for automated multi-channel segmentation to calculate partial volumes of grey and white matter (FSL FAST; Supplemental Table 2) (Zhang et al., 2001).

### 2.3 MRS analysis

Absolute metabolite concentrations were obtained relative to an unsuppressed water spectrum acquired from the same VOI. The transverse relaxation times (T2) of tissue water and percent CSF contribution to the VOI were obtained by fitting the integrals of the unsuppressed water spectra acquired in each VOI at different TE values with a biexponential fit (Piechnik et al., 2009), with the T2 of CSF fixed at 565 ms (Joers et al., 2018) and three free parameters: T2 of tissue water, amplitude of tissue water, and amplitude of CSF water (CSF fractions can be found in Supplemental Table 1). Absolute metabolite concentrations were calculated by utilizing the unsuppressed water and correcting for tissue water and CSF content. The T2 relaxation of tissue water was taken into account in the LCModel fitting by using each VOI's tissue water T2 relaxation estimations.

**Fig. 1:**
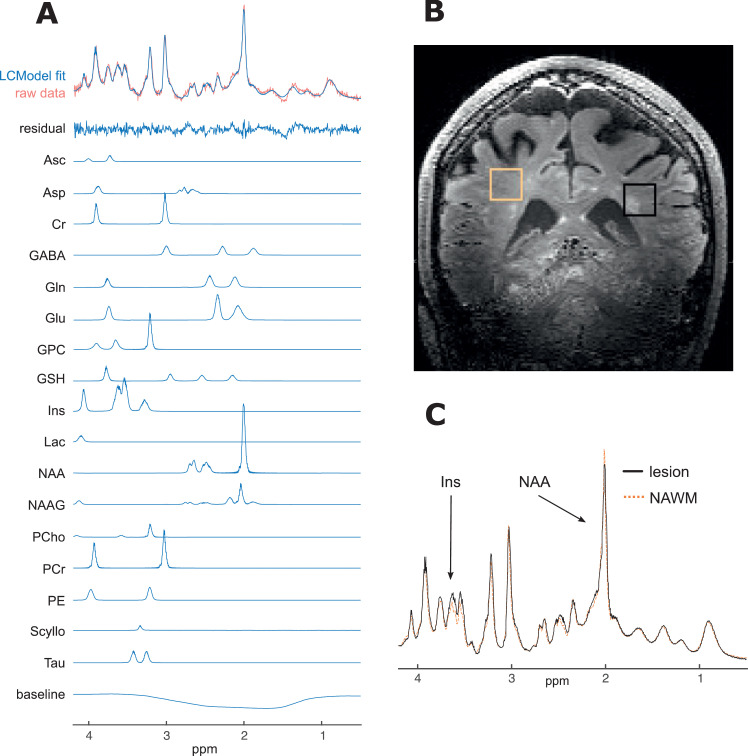
Example spectra and voxel placement from single subject. A. Raw spectrum data, LCModel fit (for average spectra and individual metabolites with CRLB < 20%), residual-error and baseline for example participant (no. 20); B. Example voxel placement (lesion outlined in black and NAWM region outlined in orange) for same subject; C. Comparison of fitted (and baseline subtracted) lesion and NAWM spectra showing differences in mI and NAA peaks, again for same subject. ppm, parts per million; Ins, *myo*-inositol; NAA, N-aspartylaspartate.

LCModel (Provencher, 2001) was used for spectral analysis and quantification. The model spectra of alanine (Ala), aspartate (Asp), ascorbate/vitamin C (Asc), glycerophosphocholine (GPC), phosphocholine (PC), Cr, phosphocreatine (PCr), γ‐aminobutyric acid (GABA), glucose (Glc), Gln, Glu, GSH, *myo*‐Ins, lactate (Lac), NAA, N‐ acetylaspartylglutamate (NAAG), phosphoethanolamine (PE), scyllo‐inositol (scyllo‐Ins) and taurine (Tau) were generated on the basis of previously reported chemical shifts and coupling constants (Govindaraju et al., 2000; Tkac, 2008) by using GAMMA/PyGAMMA simulation library of VESPA for carrying out the density matrix formalism (VErsatile Simulation, Pulses and Analysis) (Soher et al., 2011). See Fig. 1. All metabolites that underwent group analyses (Ins, NAA, NAAG and tNAA) had Cramér-Rao lower bounds (CRLBs) estimated error of metabolite quantification less than 20%.

### 2.4 Statistics

Group characteristics of age, disease duration, EDSS and minimum age of lesions were compared using permutation-based unpaired t-tests; sex and ethnicity were compared using chi-square with Yates correction. Permutation-based paired t-tests were used to compare mean T2-water estimates and measures of spectral quality. Permutation-based unpaired two-sample t-tests were used to compare lesions and areas of NAWM across groups (hypotheses 1 and 2). Permutation-based one-sample t-tests were performed to test individual lesion and NAWM metabolite concentration differences within disease groups (hypotheses 3 and 4). All group-level t-tests were computed using non-parametric permutation testing in FSL's PALM (Alberton et al., 2020; Jenkinson et al., 2012; Winkler et al., 2016). The maximum possible number of permutations (or ‘shufflings’) were used. This equated to 2048 shufflings (sign-flips) for within MS comparisons, 16 shufflings (sign-flips) for within AQP4Ab-NMOSD and 1365 shufflings (permutations) for between disease group comparisons. Note that the number of shufflings imposes an upper bound on the smallest possible p-value, that can only be as small as 1/(#permutations). Thus, for comparisons within AQP4Ab-NMOSD, the largest possible p-value is 1/16 = 0.0625. We ran statistical tests for metabolite comparisons in three batches (the fewest number that data structure and test methodology would allow) to allow for family wise error rate correction. The three batches were: permutation-based unpaired t-tests for MS and AQP4Ab-NMOSD comparisons, permutation-based one-sample *t*-test for AQP4Ab-NMOSD lesion versus NAWM differences and permutation-based one-sample t-tests for MS lesion versus NAWM differences. In order to test our directionally specific hypotheses, all *a priori* group-level t-tests were one-tailed, however all one-tailed t-tests were also run in the unexpected (opposite) direction and corrected for both directions (this is statistically equivalent to a two-tailed t-tests, but allows for meaningful comment on the uncorrected one-tailed value associated with our *a priori* hypotheses).

Tests of correlation were limited to common associations described in the literature, namely disability's association with NAA:tCr (normal appearing brain tissue & lesions) (Khan et al., 2016; Mainero et al., 2001), Ins:NAA (normal appearing white and grey matter) (Llufriu et al., 2014; Miller DH, 2014) and GABA (normal appearing grey matter) (Cawley et al., 2015), and disease duration's association with Ins, NAA, Cr, tCr and tCho (normal appearing brain tissue) (Kirov et al., 2013). Correlations were evaluated visually and with R-square (R^2^), however due to low numbers of participants and the relatively large number of tests, p-values were not calculated except for post-hoc testing of Pearson's correlation coefficient in the case of mI:NAA vs EDSS (See supplemental figure 3). Pearson's correlations performed in R (R Core Team, 2017; Wickham, 2016).

## 3 Results

Patient characteristics are listed in Table 1. Three of the four AQP4Ab-NMOSD participants were Afro-Caribbean and one was Asian, in line with the non-Caucasian predominance in this disease. One MS participant was Afro-Caribbean, the remainder were Caucasian. All lesions studied were hyper-intense on the FLAIR image and hypo-intense on the T1 weighted image. The minimum age of the brain lesions ranged from 132 days to 9 years.

**Table 1:** Demographics and clinical features.

	MS	NMO	*All*
Total n	11	4	15
Age, yrs, median (min–max)	43 (28–60)	38 (24–40)	40 (24–60)
Female, *n* (%)	7 (63.6)	3 (75)	10 (66.7)
Race			
Afro-caribbean (%)	1 (9.1)**	3 (75)**	4 (26.7)
Caucasian (%)	10 (90.9)**	0 (0)**	10 (66.7)
Asian (%)	0 (0)**	1 (25)**	1 (6.7)
Disease duration, yrs, mean (sd)	6.8 (5.5)	8.9 (3.2)	7.3 (5.1)
EDSS, median (min-max)	2 (0–6)	3.75 (0–6)	2.5 (0–6)
Minimum age of lesion, months, mean (sd)	36 (33.3)*	86.6 (27.1)*	49.5 (38.9)
On treatment, *n* (%)	8 (72.7)	4 (100)	12 (80)

* *p* < 0.05 for MS vs NMO permutation-based *t*-test comparison.

** *p* < 0.05 for MS vs NMO chi-square comparison (with Yates correction).

### 3.1 MRS quality metrics

We first wanted to ensure that there were no systematic differences in the quality of the LCModel fit between the NAWM and lesion VOIs. Reported CRLB estimates were generally low in all cases, however the number not reaching our inclusion threshold of CRLB <20% is indicated for each metabolite in Supplemental Tables 3A and B. We then wished to ensure that there was no significant difference in the quality of fit between NAWM and lesion groups. Statistical analyses demonstrated no differences, when tests were corrected for multiple comparisons (paired *t*-test p-values, α = 0.05, Bonferroni threshold = 0.05/24 = 0.0021; 0.0021 < *p* < 0.05: Asp-*p* = 0.040; *p* > 0.05 for all other metabolites; see Supplemental Table 3A and B and Supplemental figure 2).

### 3.2 T2 differences between lesions and normal-appearing tissue are relevant for metabolite quantification

There was no difference between the LCModel estimated line-widths (Full Width Half Maximum, FWHM) and Signal-to-Noise ratios (S/N) of the spectra from the NAWM and lesioned tissue (Table 2). However, estimated T2-water relaxation time was higher in lesion voxels than for NAWM voxels, reflecting the higher free-water content in this tissue (Table 2). We therefore went on to quantify neurochemicals in MS and AQP4Ab-NMOSD lesions and NAWM spectra using T2-corrected spectra. There was no estimated T2-water relaxation time difference between disease groups for combined measures, or for lesions or NAWM voxels alone (Table 2). There was also no difference in average WM/GM partial-volume in NAWM voxels between disease groups (segmented using multi-channel FSL FAST on T2 and MPRAGE; WM mm^3^, MS vs AQP4Ab-NMOSD: 2983.5 ±259.2 vs 3081 ±169.3 (mean ±sd), *p* = 0.503; GM mm^3^, MS vs AQP4Ab-NMOSD: 370.5 ±253.4 vs 281.8 ±158.5, *p* = 0.519) (Zhang et al., 2001).

**Table 2:** Estimated T2-water, LCmodel estimated FWHM and SNR.

	Lesion			NAWM		
	*MS*	*NMOSD*	*Combined*	*MS*	*NMOSD*	*Combined*
T2 water (ms)	43.3 (2.7)**	44.7 (2.7)	43.7 (2.8)*	40.1 (2.9)**	39.3 (1.6)	39.9 (2.6)*
FWHM (ppm)	0.03 (0.004)	0.03 (0.005)	0.03 (0.004)	0.03 (0.004)	0.03 (0.002)	0.03 (0.003)
S/N	25.9 (4.8)	25 (7.3)	25.7 (5.6)	28.8 (4.3)	27 (2.9)	28.3 (4)

FWHM, full width half maximum.

S/N, signal to noise ratio.

Permuted paired *t*-test, Lesion vs. NAWM: *, *p* < 0.01; **, *p* < 0.05.

### 3.3 Differences in neurochemical profiles between NAWM and lesions in MS

**Fig. 2:**
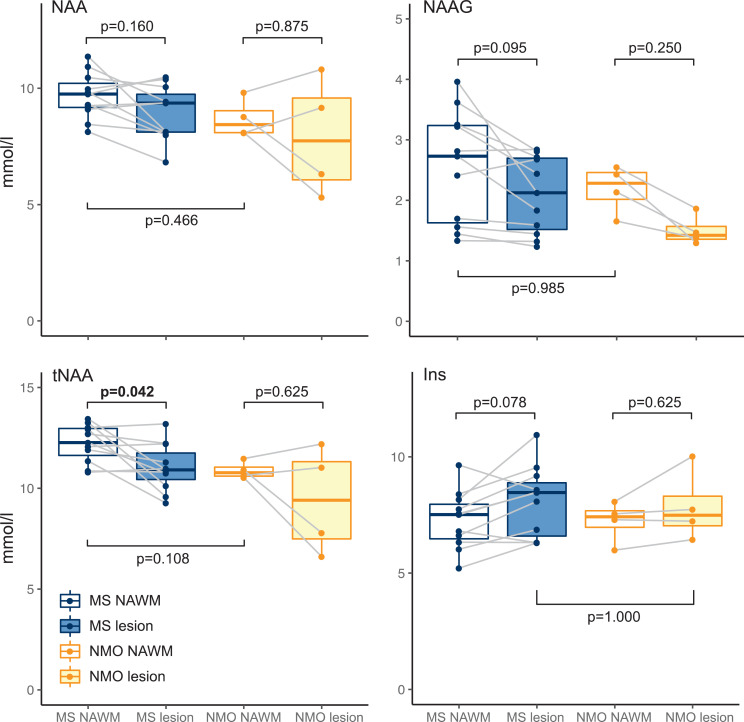
Individual metabolite comparison boxplots. Boxplot representations of metabolite comparisons. p-values generated with bidirectional permutation-based t-tests. MS, multiple sclerosis; NMO, AQP4Ab-positive neuromyelitis optica spectrum disorder; NAWM, normal appearing white matter; L, lesion. (Figure produced with R ggplot2 package; Wickham, 2016).

Next, we investigated neurochemical differences between lesions and NAWM. In the MS group absolute tNAA was lower in lesions compared to NAWM ([NAWM tNAA] – [lesion tNAA]: 1.21 ±1.31 mmol/L (mean ± SD); permutation-based one-sample t(10)=2.91, *p* = 0.042, Cohen's *d* = 0.878). We then wanted to investigate whether this difference in tNAA was driven by a decrease in NAAG, or NAA, or both. There was a trend towards lower NAAG in lesions compared to NAWM ([NAWM NAAG] – [lesion NAAG]: 0.46 ±0.57 mmol/L; permutation-based one-sample t(10)=2.52, *p* = 0.095, Cohen's *d* = 0.760), and no significance found for NAA differences ([NAWM NAA] – [lesion NAA]: 0.75 ±1.13 mmol/L; permutation-based one-sample t(10)=2.10, *p* = 0.160, Cohen's *d* = 0.634) (Fig. 2 and Table 3A). Again, there was a trend towards greater *myo*-Inositol in MS lesions versus NAWM ([lesions Ins] – [NAWM Ins]: 0.82 ±0.99 mmol/L; t(10)=2.62; *p* = 0.078, Cohen's *d* = 0.790; Fig. 2& Table 3A). See Supplemental Table 3 for a summary of all metabolites.

**Table 3A:** Multiple sclerosis: NAA, NAAG, tNAA and Ins.

	Concentration, mmol/L				CRLB	
*Metabolite*	*Lesion, mean (sd)*	*n*	*NAWM, mean (sd)*	*n*	*Lesion, mean (sd)*	*NAWM, mean (sd)*
Ins	8.1 (1.4)	11	7.28 (1.2)	11	2.73 (0.6)	3.09 (0.3)
NAA	8.94 (1.1)	11	9.69 (0.9)	11	2.55 (0.5)	2.36 (0.5)
NAAG	2.09 (0.6)	11	2.55 (0.9)	11	8 (2.5)	7 (2.9)
tNAA	11.03 (1.1)	11	12.24 (0.9)	11	1.91 (0.3)	1.91 (0.3)

### 3.4 No difference in neurochemical profiles between NAWM and lesions in AQP4ab-nmosd

With permutation-based t-tests of NAWM and lesion metabolite concentration differences, within the AQP4Ab-NMOSD patients, we found no difference in tNAA (note that this is *n* = 4; [NAWM tNAA]-[*lesion t*NAA]: 1.48 ± 2.06 mmol/L (mean ± sd); permutation-based one-sample t(3)=1.24, *p* = 0.625, Cohen's *d* = 0.621). When the two metabolites that contribute to tNAA were investigated individually, neither NAA nor NAAG differed between NAWM and lesions, despite NAAG being visibly greater in NAWM ([NAWM NAA]–[*lesion* NAA]: 0.79 ±1.84 mmol/L; permutation-based one-sample t(3)=0.74, *p* = 0.875, Cohen's *d* = 0.372; [NAWM NAAG]-[*lesion* NAAG]: 0.69 ±0.3 mmol/L; permutation-based one-sample t(3)=3.96, *p* = 0.250, Cohen's *d* = 1.979) (See Fig. 2). *Myo*-Inositol did not differ between NAWM and lesioned tissue ([NAWM Ins] – [lesion Ins]: −0.63 ±0.78 mmol/L; permutation-based one-sample t(3)=−1.40, *p* = 1.000, Cohen's *d*=−0.700) (see Fig. 2& Table 3B). There were thus no differences in either tNAA, NAA, NAAG or *myo*-Inositol between the lesion and NAWM groups in AQP4Ab-NMOSD.

**Table 3B:** AQP4-Ab positive neuromyelitis optica spectrum disorder: NAA, NAAG, tNAA and Ins.

	Concentration, mmol/L				CRLB	
*Metabolite*	*Lesion, mean (sd)*	*n*	*NAWM, mean (sd)*	*n*	*Lesion, mean (sd)*	*NAWM, mean (sd)*
Ins	7.86 (1.3)	4	7.23 (0.8)	4	2.75 (0.4)	3 (0)
NAA	7.9 (2.2)	4	8.69 (0.7)	4	2.5 (0.5)	2.5 (0.5)
NAAG	1.5 (0.2)	4	2.19 (0.3)	4	9.25 (2.9)	7.5 (2.7)
tNAA	9.4 (2.3)	4	10.89 (0.4)	4	2 (0)	1.75 (0.4)

NAWM, normal appearing white matter; CRLB, Cramér–Rao lower bound.

**Box 1:** Hypotheses.

Hypotheses	
**1**	Ins in lesions: MS > NMOSD
**2**	tNAA in NAWM: NMOSD > MS
**3a**	tNAA in MS: NAWM > lesion
**3b**	tNAA in NMOSD: NAWM > lesion
**4a**	Ins in MS: lesion > NAWM
**4b**	Ins in NMOSD: NAWM > lesion

### 3.5 Visibly greater tNAA in NAWM in MS compared with AQP4Ab-NMOSD

We then wanted to compare neurochemical concentrations between MS and AQP4Ab-NMOSD. Permutation-based unpaired two-sample t-tests were used. NAWM concentrations of NAA and NAAG were not lower in MS NAWM (AQP4Ab-NMOSD vs MS NAWM, NAA: 8.69 ±0.7 mmol/L vs 9.69 ±0.9 mmol/L; permutation-based two-sample t(13)=1.7912, *p* = 0.466, Cohen's *d* = 1.046; AQP4Ab-NMOSD vs MS NAWM NAAG: 2.19 ±0.3 mmol/L vs 2.55 ±0.9 mmol/L; permutation-based two-sample t(13)=0.7316, *p* = 0.985, Cohen's *d* = 0.427). Visual inspection of the data revealed greater tNAA in MS compared to NMOSD NAWM, but this did not reach significance (AQP4Ab-NMOSD vs MS NAWM tNAA: 10.89 ±0.4 mmol/L vs 12.24 ±0.9 mmol/L; permutation-based two-sample t(13)=2.7458, *p* = 0.108, Cohen's *d* = 1.603).

*Myo*-Inositol was not greater in MS versus AQP4AB-NMOSD lesions (MS vs AQP4Ab-NMOSD Ins: 8.1 ±1.4 mmol/L vs 7.86 ±1.3 mmol/L; two-sample t(13)=0.268, *p* = 1.000, Cohen's *d* = 0.157) (Fig. 2).

For completeness, we tested for a significant interaction of site (NAWM / lesion) by group (MS / AQP4Ab-NMOSD) for NAA, NAAG, tNAA and Ins (including our *a priori* expectations regarding the direction of difference) and this was non-significant for all comparisons (all *p* > 0.9).

### 3.6 Ins:NAA correlates with clinical score in AQP4Ab-NMOSD

Finally, we wished to investigate whether there were any relationships between our metabolites of interest and disease scores (See supplemental figure 3). Of relationships previously reported in the literature, we demonstrated a striking linear correlation between NAWM Ins:NAA and EDSS for *both* MS and AQP4Ab-NMOSD (MS: R^2^=0.22, AQP4Ab-NMOSD: R^2^=0.91, respectively; R^2^=0.30 combined). Post-hoc tests, uncorrected for multiple comparisons, showed that this was significant for the pooled data (AQP4Ab-NMOSD + MS NAWM Ins:NAA ∝ EDSS: r(13)=0.55, *p* = 0.033) and for AQP4Ab-NMOSD alone (AQP4Ab-NMOSD NAWM Ins:NAA ∝ EDSS; r(2)=0.95; *p* = 0.048), but not for MS alone (MS NAWM Ins:NAA ∝ EDSS: r(9)=0.47, *p* = 0.141).

We additionally explored the relationship between Ins:NAA, disease group (MS / AQP4Ab-NMOSD) and MRS site (lesion / NAWM) with post-hoc two-tailed t-tests (uncorrected for multiple comparisons). We found that lesion Ins:NAA was significantly greater than NAWM Ins:NAA in MS (MS lesion Ins:NAA vs NAWM Ins:NAA: 0.91 ± 0.10 vs 0.76 ±0.12; one-sample t(10)=5.42, *p* = 0.001) but for all other comparisons was non-significant.

## 4 Discussion

This study was performed in order to investigate the patterns of neurochemical changes in lesioned tissue and in normal appearing white matter in multiple sclerosis (MS) and aquaporin-4 antibody positive neuromyelitis optica spectrum disorder (AQP4Ab-NMOSD). To do this, we acquired Magnetic Resonance Spectroscopy (MRS) data using a seven tesla (7T) MRI system, with the inherent benefit of increased spectral dispersion. We demonstrated significant differences in water T2 relaxation times between lesions and NAWM, which we corrected for in subsequent analyses.

Using this corrected data, as expected, Total NAA (tNAA) was lower in MS lesions compared to MS NAWM with a trend towards greater *myo*-Inositol in MS lesions compared to MS NAWM, in line with historical MRS studies that attribute these features respectively to the axonal loss and gliosis seen in MS lesions (Arnold et al., 1992; Davie et al., 1994). We demonstrated no differences between lesion and NAWM in AQP4Ab-NMOSD patients, and no differences in either NAA or *myo*-Inositol between MS and AQP4Ab-NMOSD lesions. We did show, however, a relationship between NAA:Ins, commonly thought to be a marker of neuronal loss and gliosis, and clinical disability score, in both groups combined and AQP4Ab-NMOSD alone (Llufriu et al., 2014; Miller DH, 2014).

### 4.1 Optimised MRS allowed us to address important confounds

A number of parameters were optimised to acquire our 7T MRS spectra. The STEAM sequence was chosen despite the loss of half of the available signal to minimize relaxation effects using a short echo time. Transverse relaxation differences between lesion sites and NAWM at longer TEs have the potential to confound the quantification of metabolite concentrations.

The achieved spectral quality (high spectral resolution, SNR, efficient water suppression, and a distortionless baseline) allowed reliable quantification of 17–18 metabolites in periventricular white matter (VOI = 15 × 15 × 15 mm^3^) using LCModel analysis. T2-water relaxation times were significantly higher in lesioned tissue as confirmed in previous studies (Laule et al., 2007b, 2007a), and quantifies what one would expect given their features on T2-weighted images where lesions are identified clinically by their bright (hyperintense) appearance.

We avoided over-reliance on metabolite ratios (e.g. tNAA:tCr) and corrected for multiple T2-relaxation components. The former is of particular importance within neuroinflammatory conditions because a parallel loss of tCr in damaged tissue may conceal tNAA loss when expressed as tNAA:tCr (Davies et al., 1995). Accurate absolute quantification of metabolites, powerful in their own right, also allow for meaningful interpretation of metabolite ratios where applied.

### 4.2 NAA-G may drive lower tNAA in MS lesions versus NAWM

A decrease in NAA in MS lesions has long been described (Arnold et al., 1992; Davie et al., 1994). However, a number of questions have remained to be conclusively answered about this finding, which we can begin to address here.

Our optimised MRS methodology allowed us to accurately quantify and separate tNAA into its constituent parts: NAA and NAA-G. Although these two neurochemicals have similar molecular structures, meaning that they are hard to distinguish using MRS, they have distinct functional roles. The role of NAA is not entirely clear, but is a reflection of neuronal mitochondrial function, and has been hypothesised to have a role in myelination (Birken and Oldendorf, 1989; Moffett et al., 2007). NAAG, the most abundant peptide in the central nervous system, is found in both neurones and glia, acts as both a neurotransmitter and a glutamate reservoir, and is higher in white matter compared to grey matter (Chiew et al., 2018; Neale et al., 2000). NAAG in MS NAWM may reflect glial cell number and may also have a neuroprotective role via its ability to activate the metabotropic glutamate receptor, mGluR. As discussed further under Section 4.4 below, it is possible that higher NAWM versus lesion tNAA is a reflection of *up-regulated* NAAG in NAWM as well as loss of NAAG (and NAA) in lesions and could be interpreted as a response to disease activity (Vrenken et al., 2005). The statistical significance of MS NAWM NAA and NAAG differences presented in the results is conservative, that is, it is corrected for multiple comparisons and corrected for both directions of change. When we inspect the unidirectional (one-tailed) permutation-type *t*-test (still corrected for multiple metabolite comparisons) aligned in direction with our *a priori* hypothesis, the lower NAAG in lesions achieves a p-value of 0.049 and adds further weight to the NAAG upregulation argument expounded above.

### 4.3 No differences in NAA or *myo*-inositol between lesions and NAWM in AQP4Ab-nmosd patients

Within our four AQP4Ab-NMOSD patients, no differences were found between lesion and NAWM sites for NAA, tNAA or Ins. Interpretation of these results is of course difficult given the small number of AQP4Ab-NMOSD patients in this study. However, to our knowledge there is only one other study describing AQP4Ab-NMOSD MRS findings in central nervous system lesions and that study focuses solely on the spinal cord (Ciccarelli et al., 2013). Ciccarelli and colleagues found lower *myo*-Inositol in AQP4Ab-NMOSD lesions relative to MS lesions which in turn had lower concentrations than healthy controls, and they hypothesised that this demonstrated astrocyte loss. There are no brain studies of AQP4Ab-NMOSD lesions versus NAWM for appropriate comparison and assumptions about AQP4Ab-NMOSD brain lesion metabolites from spinal cord data should be made with caution, especially given that no AQP4Ab-NMOSD NAWM site was sampled in Ciccarelli's study and no healthy control group was sampled in ours. It must also be noted that both ours and Ciccarelli's studies have low numbers of AQP4Ab-NMOSD participants (4 and 5, respectively).

### 4.4 No evidence for MS and AQP4Ab-NMOSD metabolite differences

Our *a priori* assumptions about AQP4Ab-NMOSD and MS lesion and NAWM site differences were not confirmed. Conversely, an unexpected difference noted in plotted data suggested tNAA was greater in MS compared to AQP4Ab-NMOSD NAWM, but this did not reach significance. Low tNAA in MS in contrast to AQP4Ab-NMOSD NAWM is usually offered as support for the hypothesis that MS pathogenesis involves a chronic extra-lesional neurodegenerative processes that is absent from AQP4Ab-NMOSD pathology (Huda et al., 2019). However, this finding has been challenged by Vrenken et al. who found that the only significant difference between healthy controls and MS NAWM was a difference in NAAG (and not tNAA or NAA), and that NAAG wasn't *lower* in NAWM of MS patients but was instead *raised* compared to healthy controls (Vrenken et al., 2005). This explanation better fits with our data and is supported by a recent study of healthy participants, in whom absolute tNAA NAWM concentrations are lower than in MS NAWM here, and more in line with our AQP4Ab-NMOSD tNAA NAWM values (~8.7 mmol/L for 20–60 year olds) (Ding et al., 2016).

Again, contrary to our initial hypothesis, we did not find greater *myo*-Inositol in MS lesions compared with AQP4Ab-NMOSD lesions. It is not clear why this might be, and interpreting a null result in an n of 4 should be treated with caution, but it may be that this reflects the known pathology of some chronic AQP4Ab-NMOSD lesions where a period of gliosis supervenes over the astrocytic death of the acute lesion stage (Lucchinetti et al., 2014). MRS *myo*-Inositol has been previously suggested as a potential differentiator for MS and AQP4Ab-NMOSD because astrocytes are reduced pathologically in AQP4Ab-NMOSD and increased in MS (Geraldes et al., 2018), but this assumption rests on data from the only study besides ours to have performed MRS in AQP4Ab-NMOSD lesions, and that study was in spinal cord lesions not brain lesions (Ciccarelli et al., 2013).

One explanation for the different *myo*-Inositol concentrations between Ciccarelli *et al*'s study and the results presented here is that we have studied lesions of different ages: our study examined older lesions more likely to be in the gliotic stage (4–24 months vs 43–116 months). It is also possible that spinal cord lesions are in general more destructive than brain lesions, leading to astrocyte loss, whereas brain lesions are commonly more subtle, non-demyelinating, non-necrosing and sometimes reversible (indeed, historically, brain lesions were thought to be atypical of AQP4Ab-NMOSD)(Lucchinetti et al., 2014; Pittock et al., 2006). Finally, AQP4Ab-NMOSD lesions in the cord are centred on grey matter, while those in the brain are found primarily in white matter, which may be an important factor in gliosis (Lucchinetti et al., 2014).

Reassuringly, we found no difference in average WM/GM partial-volume in NAWM voxels between diseases. However, lesion volume estimates were on average greater in AQP4Ab-NMOSD compared to MS (MS vs AQP4Ab-NMOSD, 466mm^3^ ±365 vs 897mm^3^ ±442, permutation-type *t*-test: *p* = 0.06; see Supplemental Table 2) meaning NAWM contribution within an MRS *lesion* voxel will likely impact on metabolite concentrations and should be taken into account when interpretating AQP4Ab-NMOSD and MS lesion voxel comparisons (hypothesis 1) and within MS lesion and NAWM differences (hypothesis 4a).

Whether certain metabolic differences (e.g. NAAG or Ins), within or outside of lesions (or combined) can be used to differentiate MS and AQP4Ab-NMOSD has been the aspiration of many neuroinflammatory MRS studies and although our data holds no firm conclusions on this, we hope it aids hypothesis development in future studies.

### 4.5 Ins:NAA correlates with disability

We also found a correlation between Ins:NAA and disability (as assessed by EDSS), which was significant on post-hoc testing for both disease groups combined and for AQP4Ab-NMOSD alone (Supplemental figure 3). This correlation suggests that higher Ins:NAA relates to worse clinical score. Consistent with this finding, NAWM Ins:NAA has been shown in one study of MS patients to predict subsequent clinical disability, with higher Ins:NAA predicting greater EDSS score increase (Llufriu et al., 2014). It has been hypothesised that the CNS immune process in MS leads to an increase of *myo*-inositol and the neurodegeneration causing long-term disability results in reduced NAA, hence Ins:NAA is greater in more destructive and longer-lasting disease (Llufriu et al., 2014; Miller DH, 2014). We found a similar association of Ins:NAA and EDSS across the whole group, but this was primarily driven by the strong relationship between Ins:NAA and EDSS in AQP4Ab-NMOSD NAWM (albeit in *n* = 4). This is surprising, as the received wisdom is that AQP4Ab-NMOSD NAWM is relatively free from damage, at least outside the optic nerve and cortico-spinal tracts (Aboul-Enein et al., 2010; Bichuetti et al., 2008; De Seze et al., 2010; Matthews et al., 2015). However, even if only small differences in NAWM NAA and *myo*-Inositol occur, provided the differences decrease in magnitude with increasing EDSS, Ins:NAA will increase proportionally with EDSS. In our four AQP4Ab-NMOSD patients we found no-change or a slight increase in NAA with EDSS (i.e. no evidence of neuronal loss in NAWM in line with existing literature), along with a proportionally greater increase in Ins.

### 4.6 Limitations

Our study sought to compare AQP4Ab-NMOSD and MS neurochemicals using MRS, but has some limitations.

Firstly, no healthy control population was used as a comparator for NAWM brain voxels. Instead, patients’ own NAWM was used as a control site to compare with lesion sites. This approach was chosen as we wished to determine differences between lesioned and non-lesion tissue in our groups.  It also allowed us to avoid the difficulties of precisely matching voxel location between participants and needing to control confounding factors such as age, gender, ethnicity and medication history, which can have a substantial effect on neurochemical levels. As such, however, it is impossible to be sure whether the differences between lesioned and non-lesioned tissue shown here are driven by pathological changes in lesions or in non-lesioned tissue, or both, except through comparison with previously published values, which have invariably used different techniques and assessed different anatomical locations.

It is also possible that a structured difference in sampling location either by disease or voxel type (lesion/NAWM) might have biased the findings, given that we know MRS measures differ slightly across different lobes of the brain (Ding et al., 2016).  However, there were no systematic differences in voxel location between lesioned and non-lesioned tissue in either of the groups studied, making this potential source of bias less concerning when interpreting our results.

Secondly, there is MRS and MRI diffusion imaging evidence of mirror changes (tNAA:tCr and apparent diffusion coefficient, respectively) that occur contralaterally to sites of lesions in multiple sclerosis (Stefano et al., 1999; Werring et al., 2000). These could conceivably reduce the magnitude of lesion versus NAWM differences investigated here, or hide them altogether.

Thirdly, lesions were all at least 3 months old (i.e. chronic) at the time of assessment. This makes our results comparable with most but not all previous MS MRS lesion studies but means little insight can be gained into changes with lesion progression.

Fourthly, AQP4Ab-NMOSD disproportionately affects Afro-Caribbean individuals, whereas MS is commonly considered a disease of Caucasians. As such, matching ethnicity is difficult for studies comparing these two patient groups. It is not clear what effect, if any, this would have on the data. Whether a difference in disability between diseases influences the observed NAWM tNAA differences could also be evaluated in larger samples.

Finally, our participant numbers were low, especially for our AQP4Ab-NMOSD group (*n* = 4) and conclusions regarding metabolic differences within AQP4Ab-NMOSD and between diseases are impossible to validate. However, AQP4Ab-NMOSD is extremely rare and thus we believe these data remain of utmost importance. In Denmark, which has one of the highest reported incidences of NMOSD, there are less than 4 people diagnosed with NMO per 1000,000 per year. UK-wide prevalence of NMOSD is unknown, but is likely less than 20 per million (Hor et al., 2020). Around 60% of these will have brain lesions at some point during their disease course (Pittock et al., 2006). This, in concert with the restrictions placed on 7T scanning (especially relevant in a chronically co-morbid population), the practicalities of moving often highly disabled individuals into scanners, and the problem of tolerating extended periods in the MRI scanner for participants who commonly suffer high levels of chronic pain (Kanamori et al., 2011), meant that only a few of our small national cohort could be scanned. As such results are presented with effect sizes and thus will be readily incorporated into future meta-analyses.

## 5 Conclusion

Here we present results from an early, comprehensive metabolite profile of MS and AQP4Ab-NMOSD chronic lesions and normal appearing white matter acquired using magnetic resonance spectroscopy (MRS) at 7T. The study utilises an optimised methodology, including correction for multiple T2-water relaxation times, and our results are broadly in line with previous MRS studies in neurodegerative conditions, but serve to highlight some under-explored subtleties in MRS profiles, such as the absence of *myo*-Inositol concentration differences in AQP4Ab-NMOSD brain lesions versus NAWM and the influence of NAAG differences between lesions and normal appearing white matter. We hope that the technique described here will be highly relevant for future 7T MRS studies of this sort.

## Author statement

**George Tackley** – Conceptualization, Methodology, Software, Formal analysis, Investigation, Data Curation, Writing - Original Draft, Writing - Review & Editing, Visualization

**Yazhuo Kong** – Conceptualization, Methodology, Investigation, Writing - Original Draft, Writing - Review & Editing, Supervision, Funding acquisition

**Rachel Minne** – Software, Data Curation, Writing - Review & Editing

**Silvia Messina** – Writing - Review & Editing

**Anderson Winkler** – Methodology, Formal analysis, Writing - Review & Editing

**Ana Cavey** – Resources, Writing - Review & Editing

**Rosie Everett** – Resources, Writing - Review & Editing

**Gabriele C DeLuca** – Resources, Writing - Review & Editing

**Andrew Weir** – Resources, Writing - Review & Editing

**Matthew Craner** – Resources, Writing - Review & Editing

**Irene Tracey** – Resources, Writing - Review & Editing, Supervision, Project administration

**Jacqueline Palace** – Conceptualization, Methodology, Resources, Writing - Review & Editing, Supervision, Project administration, Funding acquisition

**Charlotte J Stagg** – Conceptualization, Methodology, Resources, Writing - Original Draft, Writing - Review & Editing, Supervision, Project administration, Funding acquisition

**Uzay Emir** – Conceptualization, Methodology, Software, Formal analysis, Investigation, Resources, Writing - Original Draft, Writing - Review & Editing

## Data statement

Due the clinically sensitive nature of the data it is have not been made freely available. However, should you or your organisation have an interest in acquiring this data for the purpose of furthering the understanding of multiple sclerosis and neuromyelitis optica, please get in touch with the corresponding author.

## Declaration of Competing Interest

GT reported no declarations of interest.; YK reported no declarations of interest.; RM reported no declarations of interest.; SM reported receiving travel grants from Biogen, Novartis, Bayer, Merck & Co, Roche, and Almirall and honorarium from Biogen for advisory work.; AW reported no declarations of interest.; AC reported no declarations of interest.; RE reported no declarations of interest.; GDL is supported by the NIHR Biomedical Research Centre (BRC), Oxford and has research funding from the Oxford BRC, MRC(UK), UK MS Society, and National Health and Medical Research (Australia). GD has received travel expenses from Bayer Schering, Biogen Idec, Genzyme, Merck Serono, Novartis, American Academy of Neurology, and MS Academy, and honoraria as an invited speaker for Novartis, American Academy of Neurology, and MS Academy.; AW reported no declarations of interest.; MC reported no declarations of interest.; IT reported no declarations of interest.; JP reported no declarations of interest.; CS reported no declarations of interest.; UE reported no declarations of interest.

## Appendix Supplementary materials

Image, application 1
