# Supplementary material for: An In-vivo 1H-MRS short-echo time technique at 7T: Quantification of metabolites in chronic multiple sclerosis and neuromyelitis optica brain lesions and normal appearing brain tissue
Source: Neuroimage. 2021 Sep;238:118225. doi: 10.1016/j.neuroimage.2021.118225 (PMC7611458; doi:10.1016/j.neuroimage.2021.118225)
Supplement: Supplementary file 1 [file mmc1.docx]

# Supplemental Data

**A note on participant numbering:**

Participants were given numerical ID numbers incrementally. Participants 1 through to 5 were pilots. Participant 8 was scanned over two sessions (and was thus scan number 8 and 10, but unique participant number 8). Participant 14 was excluded from the analyses after fulfilling secondary progressive multiple sclerosis diagnostic criteria.

**Supplemental figure 1**. Voxel placement

Numbers to the left of figures indicate participant ID number.

**Supplemental table 1**. CSF fraction (estimated with biexponential fit of unsuppressed water spectra)

| **Participant number** | **Voxel type** | **Disease** | **Estimated CSF fraction (%)** |
| --- | --- | --- | --- |
| 6 | NAWM | MS | 4.5 |
| 7 | NAWM | MS | 3.9 |
| 10 | NAWM | MS | 3.1 |
| 13 | NAWM | MS | 6.8 |
| 15 | NAWM | MS | 3.2 |
| 16 | NAWM | MS | 1.6 |
| 17 | NAWM | MS | 2.9 |
| 18 | NAWM | MS | 2 |
| 20 | NAWM | MS | 11 |
| 21 | NAWM | MS | 4.3 |
| 22 | NAWM | MS | 2.1 |
| 9 | NAWM | NMO | 8.4 |
| 11 | NAWM | NMO | 3.7 |
| 19 | NAWM | NMO | 2.1 |
| 23 | NAWM | NMO | 4.2 |
| 6 | Lesion | MS | 5.6 |
| 7 | Lesion | MS | 2.6 |
| 8 | Lesion | MS | 2.9 |
| 13 | Lesion | MS | 3.7 |
| 15 | Lesion | MS | 4 |
| 16 | Lesion | MS | 3.1 |
| 17 | Lesion | MS | 2.6 |
| 18 | Lesion | MS | 2.8 |
| 20 | Lesion | MS | 12.5 |
| 21 | Lesion | MS | 5.3 |
| 22 | Lesion | MS | 1.9 |
| 9 | Lesion | NMO | 4 |
| 11 | Lesion | NMO | 9.2 |
| 19 | Lesion | NMO | 3.2 |
| 23 | Lesion | NMO | 7.1 |

**Supplemental table 2**. Lesion partial volume estimates (T2-weighted image manual segmentation)

| **Participant number** | **Disease*** | **Lesion volume mm^3^** |
| --- | --- | --- |
| 6 | MS | 262 |
| 7 | MS | 266 |
| 8 | MS | 782 |
| 13 | MS | 593 |
| 15 | MS | 1380 |
| 16 | MS | 70 |
| 17 | MS | 524 |
| 18 | MS | 197 |
| 20 | MS | 389 |
| 21 | MS | 442 |
| 22 | MS | 221 |
| 9 | NMO | 805 |
| 11 | NMO | 537 |
| 19 | NMO | 708 |
| 23 | NMO | 1539 |

* MS < NMO lesion volume, permutation-type unpaired t-test p=0.06

NMO, AQP4Ab positive neuromyelitis spectrum disorder

**Supplemental table 3A**. Multiple sclerosis metabolite profile (n=11)

**Supplemental table 3B**. AQP4-Ab positive neuromyelitis optica spectrum disorder metabolite profile (n=4)

NAWM, normal appearing white matter; CRLB, Cramér–Rao lower bound

**Supplemental figure 2**. Metabolite profiles for multiple sclerosis and AQP4Ab positive neuromyelitis optica

Box and whisker plots of metabolites for MS and AQP4Ab-NMOSD (NMO; min, lower-quartile, median, upper-quartile, max; outliers are >1.5 x interquartile range beyond lower or upper quartiles).

MS, multiple sclerosis; NMO, AQP4-antibody positive neuromyelitis optica; NAWM, normal appearing white matter

**Supplemental figure 3.** Correlation with clinical metrics

EDSS, Expanded Disability Status Scale; NAWM, normal appearing white matter
